# Effect of the software binning and averaging data during microcomputed tomography image acquisition

**DOI:** 10.1038/s41598-019-46530-z

**Published:** 2019-07-22

**Authors:** Simone Peixe Friedrichsdorf, Victor Elias Arana-Chavez, Paolo Maria Cattaneo, Rubens Spin-Neto, Gladys Cristina Dominguez

**Affiliations:** 1 Department of Orthodontics and Pediatric Dentistry, School of Dentistry, University of São Paulo (USP), São Paulo, Brazil; 2 Department of Biomaterials and Oral Biology, School of Dentistry, University of São Paulo (USP), São Paulo, Brazil; 3 Department of Dentistry and Oral Health, Section of Orthodontics, Aarhus University, Aarhus C, Denmark; 4 Department of Dentistry and Oral Health, Section for Oral Radiology, Aarhus University, Aarhus C, Denmark; 5 Department of Orthodontics and Pediatric Dentistry, School of Dentistry, University of São Paulo (USP), São Paulo, Brazil

**Keywords:** Three-dimensional imaging, Computed tomography

## Abstract

This study describes the effect of the software binning and data averaging during micro CT volume acquisition, on the assessment of root resorption volumes. The mesial roots (n = 9), after orthodontic tooth movement during 14 days, were scanned, using a micro CT system (9 µm/pixel). All roots were reconstructed and the volumes of the resorption lacunae evaluated. The height and width of the pixels vary according to the parameters (A1, A2, A3, A4, A5, A6, A7, A8, A9) used during the scan. In the root #1 the mean volumes of resorption were similar in A4 and A7; in the root #2 there was no similarity in the mean volumes of resorption in any of the parameters; in root #3 only A4 presented mean volume different from zero (3.05 × 10°). In the root #5, the A1 and A7 presented similar mean volumes and in the A6 and A9 presented near mean volumes. In the root #9 the A1, A4, and A7 presented similar mean volumes and A6 and A9 also had similar mean volumes. Significant difference was detected in the volume of resorption among the roots #2, #5 and #9 (*p* = 0.04). When analyzing delicate structures such as the roots of rats’ molars, the variation of such parameters will significantly influence the results.

## Introduction

Micro-computed tomography (micro CT) is a non-destructive technique used for the generation of cross-sections of a given material through a set of plane projections with the same physical principle of computed tomography (CT)^1^. One important advantage of micro CT is the voxel dimension between 1 and 100 μm, which is decisively suitable to preclinical applications as it is much higher than that of clinical CT^2,3^. Micro CT is able to represent 3D structures of biological samples with a high amount of detail, and it has become a common method in studies associated with mineralized tissues^4,5^. The leading application of micro CT in biology is the structural study of bones and teeth^6,7^.

The first step in micro CT processing is acquiring a digital image (image acquisition). The next step is image preprocessing with the purpose of improving the image, for example, improving contrast enhancement and noise removal. Subsequently, segmentation of the image is performed^8^. In this context, segmentation refers to the separation of different tissues from each other by extracting and classifying features^9^. Analysis of the micro CT results depends on the acquisition parameters, which may have significant effects on the results^10–15^.

In recent years, micro CT imaging has been used to investigate root resorption during orthodontic tooth movement for *in vivo*^16–19^ and *ex vivo* studies^20–22^. For such applications, one of the advantages of using such a method is that the structure of the tooth root can be mapped down to the micrometer level in three dimensions. However, there has been no study comparing some relevant image acquisition parameters in the outcomes of the root resorption evaluations. Among these parameters, data averaging and software binning could be listed as relevant.

Data averaging refers to scanning the same region multiple times, thereafter averaging the acquired signals to construct the final images. Since more scan cycles are needed, the overall image acquisition time increases proportionally. Software binning refers to the combination of pixel matrices (e.g., 2 × 2, 3 × 3, etc.…) to create one larger pixel. This combination increases the signal level measured from the larger effective sensor pixel, leading to more contrast and less noise. However, the native resolution of the images will also be lower since it would be as if the pixel and therefore the voxel sizes were larger. Since pixel binning reduces the amount of information to be transferred to the computer, the acquisition time can be slightly diminished when such a parameter is altered^23^. Currently, some studies have evaluated root resorption by microcomputed tomography. Before image acquisition, it is important to correctly set some parameters for microcomputed tomography imaging, that may influence the results. In this study, the authors have chosen data averaging and software binning because there are no previous studies about this topic in the literature. Thereby, this study describes the effect of varying two of these parameters, i.e., the software binning and data averaging during volume acquisition, on the assessment of root resorption volumes.

## Results

The overall ICC was 0.72. The individual ICCs varied; roots #2 and #3 had an ICC < 0.4; roots #1, #4 and #6 showed 0.4 ≤ ICC < 0.75; and roots #5, #7, #8 and #9 had a CHF greater than 0.75.

The height and width of the pixels varied according to the parameter used during the scan. The parameters A1, A4 and A7 presented the same width (pixel) 1032; the parameters A2, A5, A8 presented the same Width (pixel) 692; and the parameters A3, A6 and A9 presented the same width (pixel) 520. The height (pixel) changed for each parameter and in each scanned root (Table 1).

**Table 1:** Height and width of the pixels.

			A1	A4	A7	A2	A5	A8	A3	A6	A9
R1	Pixel size (µm) 264.62	Width (Pixel)	1032			692			520		
		Height (Pixel)	190	137	136	99	90	91	71	68	70
R2		Width (Pixel)	1032			692			520		
		Height (Pixel)	184	179	178	124	120	122	88	91	90
R3		Width (Pixel)	1032			692			520		
		Height (Pixel)	222	216	213	146	145	143	108	109	110
R4		Width (Pixel)	1032			692			520		
		Height (Pixel)	198	193	194	134	105	129	100	96	95
R5		Width (Pixel)	1032			692			520		
		Height (Pixel)	150	144	148	101	99	99	76	75	73
R6		Width (pixel)	1032			692			520		
		Height (Pixel)	141	140	139	92	95	93	71	70	70
R7		Width (Pixel)	1032			692			520		
		Height (Pixel)	185	183	180	123	124	121	92	91	92
R8		Width (Pixel)	1032			692			520		
		Height (Pixel)	207	206	206	138	139	138	105	104	103
R9		Width (Pixel)	1032			692			520		
		Height (Pixel)	190	189	186	126	127	126	95	96	96

**Figure 1:**
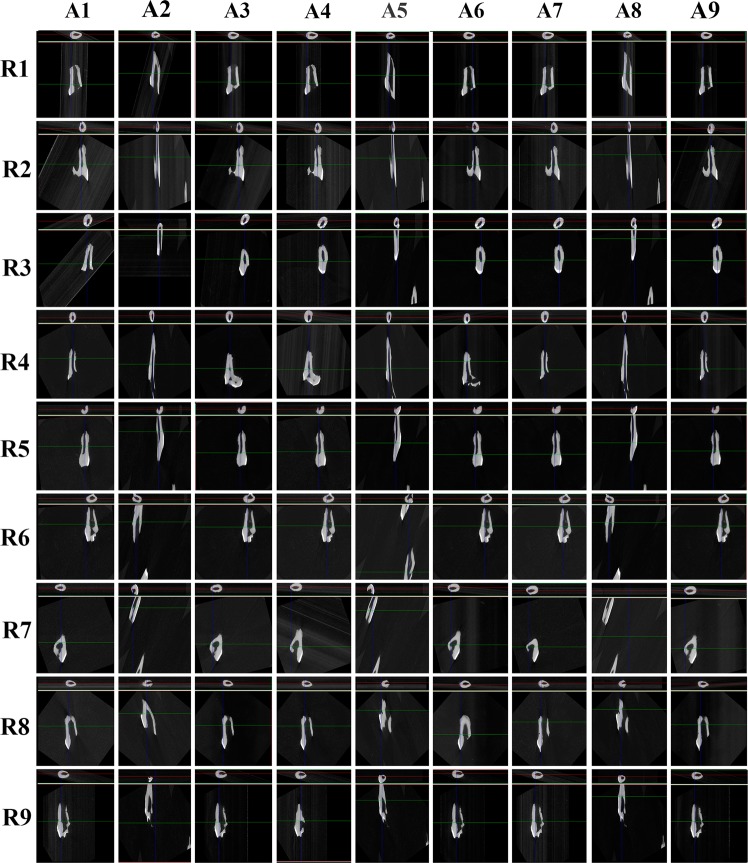
The images of roots in different parameters.

Only in roots #1 and #8 was it possible to calculate the volume of the resorption lacunae in all parameters. In root #4, it was not possible to verify the root resorption volume in parameter A5; and for roots #2, #3, #5, #6, #7 and #9 in the parameters A2, A5, and A8, it was not possible to calculate the root resorption volumes due to root elongation in the transaxial cut (Z- axis) and flattening in the coronal cut (X-axis) (Fig. 1).

In root #1, the mean volumes of resorption were similar in parameters A4 and A7 (2.07 × 10^−1^, 2.31 × 10^−1^); in root #2, there was no similarity in the mean volumes of resorption for any of the parameters; in root #3, only parameter A4 presented with a mean volume different from zero (3.05 × 10°). In root #5, the parameters A1 and A7 presented similar mean volumes (6.26 × 10^1^, 6.43 × 10^1^) and parameters A6 and A9 presented near mean volumes (1.14 × 10^1^, 1.15 × 10^1^). In root #9, the parameters A1, A4 and A7 presented with similar mean volumes (3.05 × 10^1^, 3.51 × 10^1^, 3.04 × 10^1^), and parameters A6 and A9 also had similar mean volumes (1.10 × 10^1^, 1.07 × 10^1^) (Table 2).

**Table 2:** Volumes of resorption on the roots.

root	Parameters	Volume (mm^3^)		P
		Mean	SD	
1	1	3.64 × 10^−1^	1.80 × 10^−1^	0.06
	2	5.37 × 10^−2^	3.82 × 10^−2^	
	3	0	0	
	4	2.07 × 10^−1^	1.89 × 10^−1^	
	5	0	0	
	6	0	0	
	7	2.31 × 10^−1^	1.80 × 10^−1^	
	8	4.63 × 10^−3^	4.63 × 10^−3^	
	9	0	0	
2	1	7.87 × 10^−1^	7.28 × 10^−1^	0.04
	2	#	#	
	3	6.18 × 10^−3^	6.18 × 10^−3^	
	4	3.50 × 10^−1^	3.50 × 10^−1^	
	5	#	#	
	6	3.09 × 10^−3^	3.09 × 10^−3^	
	7	3.78 × 10^−2^	3.78 × 10^−2^	
	8	#	#	
	9	0	0	
3	1	0	0	0.51
	2	#	#	
	3	0	0	
	4	3.05 × 10°	1.42 × 10^1^	
	5	#	#	
	6	0	0	
	7	0	0	
	8	#	#	
	9	0	0	
4	1	1.85 × 10°	9.85 × 10^−2^	0.51
	2	0	0	
	3	5.60 × 10^−1^	5.57 × 10^−1^	
	4	7.96 × 10^−2^	2.63 × 10^−2^	
	5	#	#	
	6	1.66 × 10°	7.50 × 10^−1^	
	7	1.20 × 10^1^	2.62 × 10°	
	8	0	0	
	9	2.89 × 10^−1^	1.59 × 10^−1^	
5	1	6.26 × 10^1^	0	0.04
	2	#	#	
	3	9.67 × 10°	3.67 × 10^−1^	
	4	7.06 × 10^1^	4.14 × 10°	
	5	#	#	
	6	1.14 × 10^1^	8.20 × 10^−1^	
	7	6.43 × 10^1^	1.03 × 10°	
	8	#	#	
	9	1.15 × 10^1^	1.79 × 10°	
6	1	5.05 × 10°	2.79 × 10°	0.27
	2	#	#	
	3	1.12 × 10^1^	1.64 × 10°	
	4	4.39 × 10^−1^	2.64 × 10^−1^	
	5	#	#	
	6	2.34 × 10°	7.83 × 10^−1^	
	7	1.49 × 10°	8.84 × 10^−1^	
	8	#	#	
	9	1.98 × 10°	1.66 × 10°	
7	1	2.21 × 10^−1^	7.72 × 10^−3^	0.27
	2	#	#	
	3	1.05 × 10^1^	9.62 × 10^−1^	
	4	8.31 × 10^−1^	7.42 × 10^−2^	
	5	#	#	
	6	6.96 × 10^−1^	1.61 × 10^−1^	
	7	3.09 × 10^−3^	0	
	8	#	#	
	9	5.27 × 10^−1^	1.08 × 10^−1^	
8	1	1.97 × 10°	4.94 × 10^−1^	0.83
	2	5.60 × 10°	1.07 × 10°	
	3	2.31 × 10°	1.04 × 10°	
	4	2.14 × 10°	2.67 × 10^−1^	
	5	9.20 × 10°	9.82 × 10^−1^	
	6	0	0	
	7	4.26 × 10°	1.81 × 10°	
	8	1.41 × 10°	4.77 × 10-^1^	
	9	1.19 × 10^1^	4.06 × 10°	
9	1	3.05 × 10^1^	7.37 × 10°	0.04
	2	#	#	
	3	9.07 × 10^0^	1.35 × 10^0^	
	4	3.51 × 10^1^	1.74 × 10^−1^	
	5	#	#	
	6	1.10 × 10^1^	9.71 × 10^−1^	
	7	3.04 × 10^1^	5.09 × 10^0^	
	8	#	#	
	9	1.07 × 10^1^	2.35 × 10^0^	

# The volume value of the resorption lacunae was not calculated.

The intragroup comparison showed that there was no statistically significant difference between the parameters in roots R1, R3, R4, R6, R7 and R8 (p > 0.05). Roots #2 and #5 had a statistically significant difference between the parameters (p < 0.05) (Table 2).

## Discussion

In the present study, it was possible to perform 3D reconstruction of the roots in all tested parameters; however, the change in the dataset size and the dimension of the datasets in pixels were modified, and, consequently, the volume of the root resorption was changed. The results of the present study suggested that increased software binning decreased pixel dimensions and reduced the volume of root resorption. Pixel size was larger when software binning was set to 1, and it was lower when software binning was set to 2, independently of the data averaging selection.

A voxel is the discrete unit of the scan volume that is the result of any tomographic reconstruction (e.g., micro CT acquisition). A voxel is also the three-dimensional representation of a pixel or, in other words, the volumetric representation of sections with a defined height, width, and thickness. The smallest voxel size available should be used for all μCT scans, although some scans are not desirable because they require longer acquisition times and generate large data sets^24^. Therefore, the correlation between voxel size and scan time should be considered^12^.

The information content of a voxel depends on the signal-to noise ratio (SNR), and this is governed by the number of incident photons and the sensitivity of the charge-coupled device (CCD) detector^12^. The total number of photons for each projection during a micro CT acquisition depends on the tube current (mA) and the integration time for each projection (ms), as well as the number of times each projection is repeated (i.e., data averaging)^12^. In the present study, data averaging increased the image acquisition time because more scan cycles are needed.

In this *in vitro* study, the variation in the acquisition parameters affected the number of projections, the size of the data set, and the resolution of the volume. The pixel size changed when the software binning was modified; however, using the same software binning with different data averaging did not affect the pixel size. The increase in the pixel size led to larger data sets due to the greater number of projections during the acquisition of the volume. The contrast of the scanned image was better when the data average and the software binning smaller. It is important to emphasize that the larger projection results in a longer acquisition time and consequently increases the radiation dose; therefore, it is important to consider it in *in*-*vivo* studies (e.g., anaesthetic time, and the effect of the radiation dose on the animals). To analyse the details of the structure (small bone structures within bone), it is suggested that a lower software binning and data averaging is selected; however, it is essential to consider the type of acquisition (*in vitro* or *in vivo*). To analyse anatomical structures (position and angulation), it might be better to use larger software binning and data averaging, and, in this way, acquisition time and data set size are smaller.

It is relevant to point out that, although all the roots included in the presented study presented with a degree of root resorption, some of the tested image acquisition parameters values caused the resorption not to be detectable. Subjectively, the main visual effect of changing the parameters was the elongation (crown-apex) and the flattening of the root (labial-lingual). When software binning 1.5 and 1, 2, 3 averaging were used, the root images seemed longer and with a smaller diameter than the other images when using software binning 1, 2 and 1, 2, 3 averaging.

Data averaging and software binning are image acquisition parameters that influence the pixel size, signal-to-noise ratio, image acquisition time and data set size. When analyzing delicate structures such as the roots of rats’ molars, the variations in such parameters will significantly influence the results. The entire volume of the root resorption decreased or was not detected when data averaging was reduced.

## Methods

Nine 10-week-old Wistar rats (250–300 g) were used in this study. During the experiment, the rats were kept inside appropriate cages in rooms with a controlled temperature (25 °C) and 12/12 h light cycles, with access to a powdered diet and water *ad libitum*. Principles of laboratory animal care (NIH publication 85–23, 1985) and the national laws on animal use were observed for the present study, which was authorized by the Ethical Committee on Ethics in the Use of Animal of the University of São Paulo (004/2016).

### Tooth movement

All procedures were carried out under general anaesthesia using an intramuscular injection of 12 ml/100 g body weight mixed ketamine hydrochloride and 6 ml/100 g body weight xylazine hydrochloride. Experimental orthodontic tooth movement was performed using a closed-coil spring that was bonded to the first upper left molar cleat with a stainless steel ligating wire (wire size: 0.008″, Morelli®, SP, Brazil). The other side of the coil spring was bonded to the upper left incisor exerting 25 cN force to mesially move the molar. The coil was maintained in the teeth for 14 days.

### Orthodontic root resorption

The rats were euthanized (by decapitation) after 14 days^25^, and the maxillary left first molars were carefully extracted and the roots were checked for integrity under a stereomicroscope. The mesial roots were separated using a diamond disc. Only the mesial roots were used in this study and they were scanned nine times using a micro CT system (μCT 40, Scanco Medical, Bruttisellen, Switzerland) at a resolution of 9 µm/pixel through 180° of rotation with 0.5° stepped increments of 2 degree; however, each time, the values of software binning (1, 1.5, 2) and data averaging (1, 2, 3) were changed (Table 3). The sample preparation and positioning were the first steps of image acquisition. In this study, the samples were aligned with the vertical axis of the scanner inside the tube. The tube and samples were maintained in the same original position during all the scans to avoid any bias in the images. The scan medium can affect the X-ray attenuation^26^. Therefore, in this study, no medium (i.e. only air in the tube) was chosen to scan the specimens. The raw data were further reconstructed using the software DataViewer (Bruker microCT, Kontich, Belgium) and the volumes of the resorption lacunae were evaluated with the software CTAn (Bruker microCT, Kontich, Belgium).

**Table 3:** Software binning and data averaging.

Parameters	Avering data	Software Binning
A1	1	1
A2		1.5
A3		2
A4	2	1
A5		1.5
A6		2
A7	3	1
A8		1.5
A9		2

**Figure 2:**
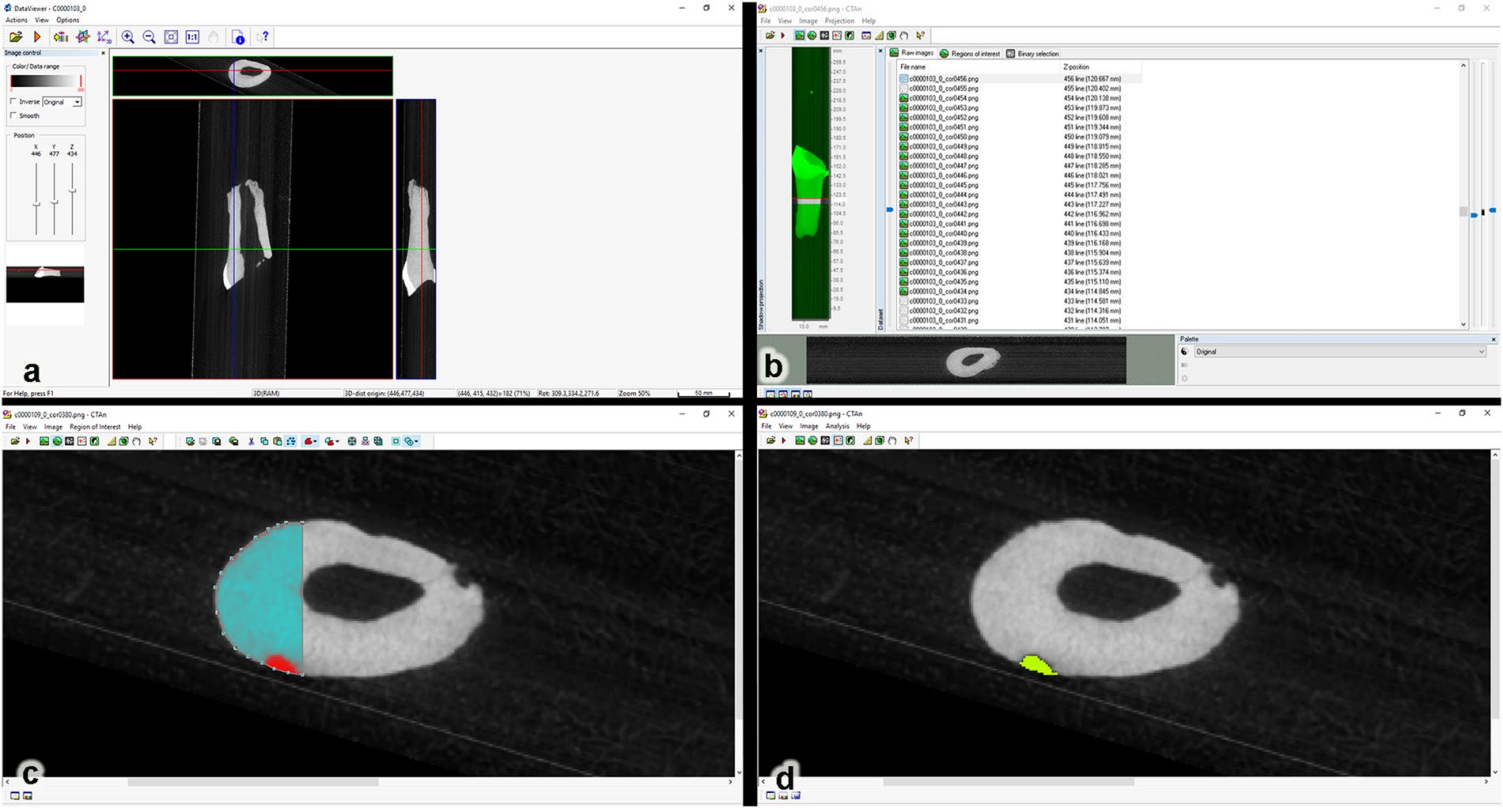
(**a**) The largest diameter was positioned parallel to the horizontal plane; (**b**) The coronal images. The largest total length (H_T_) of each root was measured, i.e., from the amelodentinal junction to the root apex, and this value was divided by 2 (H_T_/2). From the midpoint, the top (H_T_/2 + 10) and bottom (H_T_/2–10) of the root was established; (**c**) The region of interest (ROI) was established for each slice; (**d**) The 3D volume of root resorption was calculated with the morphometry tool.

Each dataset was opened with software DataViewer, that provided tools for 3D image registration and the images were rotated. The largest diameter of the roots were positioned parallel to the horizontal plane, and the coronal images were saved as a new dataset (Fig. 2a).

The volumes of the resorption lacunae were evaluated with the software CTAn. The coronal images (new dataset) were opened on CTAn software, and the largest total length (H_T_) of each root was measured, i.e., from the amelodentinal junction to the root apex. After that, this value (H_T_) was divided by 2 (H_T_/2), defining the midpoint of the root. From the midpoint, were added 10 sections for each end (H_T_/2 + 10) of the selection: 10 on the top of selection; and 10 at the bottom of selection (H_T_/2–10) of the root was established, obtaining thus 20 images (Fig. 2b).

The region of interest (ROI) was established for each slice, filling the resorption lacuna (Fig. 2c). The threshold (between 0–80) was interpolated from the dataset. The 3D volume of root resorption was calculated with the morphometry tool (Fig. 2d).

The same blind and calibrated operator performed all measurements, and every measurement was repeated two times, with a two-week interval between measurements.

### Statistical analyses

All statistical analyses were performed using SPSS Statistical Package version 24 (SPSS Inc, Chicago, IL). Descriptive statistical analysis was performed to report the results of all assessments. The Intraclass Correlation Coefficient (ICC) was calculated to assess the reliability and agreement between the measurements of the volume of root resorption. The Kruskal-Wallis Test was used for comparisons between groups. The level of statistical significance was set at 0.05.

### Ethics approval and consent to participate

All applicable international, national, and institutional guidelines for the care and use of animals were followed.
